# Annual Address before the Erie County Medical Society

**Published:** 1865-02

**Authors:** S. W. Wetmore


ART. II.—*Annual Address before the Erie County Medical Society.* By S. W. WETMORE, M. D.

*Mr. President and Gentlemen:*

The subject chosen for consideration at this our annual meeting, is that of *Practical Anatomy,* and some of its relations to *Medicine* and *Surgery.*

I do not propose to advance any new theories, or expect to teach this learned body any facts which they are not already familiar with, but, perhaps, make some suggestions which may prove of some utility to the practitioner who neglects this important branch of our profession.

As Anatomy is defined to be the science of the structures of organized bodies or living beings, it would comprise both vegetable and animal anatomy; with the former the botanist is more particularly concerned, of which I do not propose to speak.

Animal anatomy is divisable into zoological or comparative and special anatomy. It receives the former appellation when the entire series of animals is considered, comparing the same organs in the different species; and when it is restricted to the examination of one species, it is denominated special anatomy, as the anatomy of man, which is the division under consideration. A practical knowledge of human anatomy can only be acquired by dissections of the cadaver. It is descriptive anatomy only that can be obtained from text-books. The same cannot be said of pathological, regional or surgical anatomy.

For the purpose of the prosecution of the study of human anatomy, it is generally advisable to prepare the subject for the prevention of decomposition, so that the structures may retain their solidity, color and physiological conditions as much as possible. For this purpose, let us first take into consideration the best possible mode of preparing the material. From time immemorial anatomists have used various substances for this purpose, although it was not until the beginning of the last century that antiseptic and corroded preparations were introduced, and then by Francis Nichols, professor of anatomy at Oxford. This eminent professor, after having experimented with many of the metalic and alkaline preparations, among which were muriate of mercury, white oxide of arsenic, muriate of ammonia, nitrate of potash, the aluminous salts, etc., gave preference to muriate of soda, nitrate of potash, sugar house molasses, and starch in solution, to be used by injection, Since his time, various vegetable and mineral acids, creosote, the essential oils, alcohol, etc., have been in vogue. The preparations most commonly used in medical colleges at the present time is a combination of corosive sublimate, creosote and alcohol. There are none of the above articles that have the desired effect, that of prohibiting putrefaction, preventing disagreeable odors, and retaining the solidity of the tissues; but, happily, recent discoveries bring to our aid an article which renders all former preparations obsolete, I refer to the arsenite of soda, which I have had the honor of introducing into the University of Buffalo, where it has been tested for the last past three years. This is not an officinal preparation, nor have I been able to learn by whom it was first prepared, but having ascertained that such an article was in use for this purpose by some who chose to keep latent the *modus operandi* of preparing and using it, I consulted with Prof. Geo. Hadley for the purpose of investigating its composition, properties, best mode of using, etc., and after experimenting some little time, he succeeded in consummating the article sufficiently for all practical purposes, though its formula, equivalent, etc., have not as yet been ascertained. It is prepared by boiling 12 ounces crystalized carbonate of soda and 8 ounces white arsenic in 32 fluid ounces of water for one hour; dilute with water so that 40 fluid ounces contain 3 ounces of white arsenic. This is sufficient for one subject, and can be injected by means of a large syringe into the femoral artery, and through this into all parts of the system.

By experimenting I find that not less than three or four ounces of arsenic, as combined here, will answer the desired object, which under favorable and ordinary circumstances, not only acts as a disinfectant, but an antiseptic, destroying all virus, rendering the tissues firm, the muscles retaining their color and solidity, prohibiting decomposition and offensive odors; in fact it gives a sweet smell to the tissues, as our worthy professor of anatomy, Dr. Eastman, frequently remarks during his lectures—getting his olfactories near a muscle—that "it really has a sacharine odor."

It is not only peculiar but remarkable that bodies injected with this article remain free from vermin, flies having but little desire to deposit their ova, and when deposited inevitably fail to mature, the process of incubation having been destroyed probably by the poisonous effects of the arsenic. The favorable circumstances suggested above has reference to the condition of the material previous to the introduction of the antiseptic. It would not be reasonable to suppose that a subject which was partially decomposed, or in which putrefaction had already commenced could be as easily preserved as one in which the tissues had not undergone any change. Much depends not only upon the condition of the cadaver, the time elapsed since death, etc., but upon the malady which rendered life extinct.

There are many diseases and conditions of the system which promote after death the rapid decomposition of the tissues. As a general rule—excepting in diseases of the heart and air passages—those diseases which destroy life most rapidly are followed by the most rapid decomposition of the body; this depends probably upon the condition of the fluids of the body, and particularly the blood, as is frequently observed in the adynamic fevers, typhus, typhoid, and the pernicious or congestive chill, in variola, diphtheria, etc. It is also observed in *sun-stroke*, in which the cadaverous odor is perceivable oftentimes before death.

In apoplexy, the brain (the part involved,) is generally found in a broken-down, disintegrated condition, emitting an offensive, putrid smell. Many others might be mentioned in which experience has taught to be almost an impossibility to prohibit decomposition, particularly in pyæmia, and other blood poisons, unless secured early; by this is meant before the subject gets cold. I find on experimenting on animals bled to death, that if they are injected properly, and as soon after life is extinct as possible, and before the rigor mortis has appeared, that they will keep for almost an indefinite length of time even when exposed to an open and changeable air and the hot rays of the sun; the hair even remaining adherent, and not becoming detached in many months and even years. The same has been claimed for arsenic without the combination with soda, but my experiments have all proved futile, except with the article under consideration.

It would appear the greater amount of fluid, serum, blood, &c,, in the body, the greater the tendency to change of tissue; this is evinced on the one hand by dropsical subjects which are prone to a softened tissue; and on the other, by the great facility by which animals are preserved that have died of hemorrhage. This is the manner in which soldiers are embalmed; and those who die of hemorrhage, it is said, are most easily preserved, some of whom have been entombed and in wooden cases since 1861, and without material change, except a general dessication of the features.

Another great desideratum is attained by this article, which is not claimed for any other preparation, that of destroying all virus in the subject: so there is no hazard of poisoning, when the operator pricks, scratches or cuts his fingers, which is frequently the case with students in the dissecting room, who seldom pay any attention to it, and I have yet to learn of the first case of a poisonous or deleterious result. Neither is there any poisonous effects arising from the arsenic, which is claimed by some to be an objection to arsenical preparations.

After the material has been well injected for the purpose of prohibiting decomposition, &c., it should be allowed to remain from twelve to twenty-four hours, when the arteries will be found empty; they should then be filled with something designed to distend them permanently, which will facilitate the tracing and study of the smaller branches very materially. For this purpose I have been in the habit of using simple beef's tallow colored with venetian red, and injected quite hot in the same manner and place as before. If this manipulation be conducted with a proper syringe and some degree of force and dexterity it will be found to have entered even the capilaries in some instances, I have frequently seen the *vasa intestini tenuis* with all their various anastomosing loops which form a series of arches by their frequent inosculations, distended to their utmost capacity, and when viewed in a strong light by looking through the two layers of mesentery, it presents a beautiful tesselated pavement, which the artist would fail to depict.

Nor have my observations been confined to points so near the aorta. The superficialis valve which not unfrequently is of extreme tenuity, passing between the fibres, or in front of the abductor pollicies muscle and completing the superficial Palmer arch by inosculating with the termination of the ulnar, is often as beautifully distended as is creditable to the vital current.

It is also as beautifully demonstrated in the branches of the dorsalis pedis and halucis, or at the base of the brain, where the remarkable anastomosis that takes place between the branches of the internal carotid and vertebral arteries form the circle of Willis.

The distension of the arteries is almost indispensable; the same cannot be said of the veins, as they are generally concomitants to the arteries, and more or less filled with blood, and by their color can be easily designated from the arterial and nervous fillaments. They are however sometimes distended with tallow colored with Prussian blue, but the presence of their numerous valves, renders the operation precarious and generally imperfect.

Our material is now considered in readiness for the study of *practical anatomy.*

It is not my purpose here to enter into details pertaining to anatomical demonstrations, but to call the attention of the practitioner to the imperfect manner in which it is taught or acquired, and the necessity of a more thorough anatomical education.

I will venture, though Iregret to say, that this, the primary, the fundamental study, the base on which to build a medical education is the least perfected of all the various branches pertaining to the healing art. It is remarkable and surprising to see the perfect inability, the incompetency, the absence of even a vague idea of the structure of the human system which exists among some practitioners of good standing; the same can be said of many recent graduates of some of our universities. This is an opprobrium not only to the fraternity generally, but to those institutions which pass men of these qualifications. There is however another class who have a better idea of their organization, that is, they know the osseous system forms the skeleton, the frame-work of the animal, and that they help to form cavities which contain the vital parts, the nervous cavities, etc., and give attachment to muscles, ligaments, tendons and synovial membranes, but a description of a single one of them, even the origin and insertion of a muscle, saying nothing of its functions would be impossible. They are also aware that the heart is the central organ of the circulation, and that the arteries carry arterial or florid, and the veins, venous or dark blood, but how the change of color takes place, or of the circulation through the heart, or of this organ's structure in fœtal as well as adult life, consisting of valves, their names, situation, etc., they have a very imperfect knowledge. Again of the deep structures, the viscera, the arteries and their relations, the nerves, their origin. distribution and functions of which they are equally as ignorant, hence their surgical and practical anatomy must be limited. It is unnecessary in this connection to state that there is another class of students and practitioners who perfect this important study, as much as possible, and investigate its relations to medicine and surgery, some of which I propose now to notice; for this purpose we will briefly consider the *cranial nerves*, which in a pathological point of view probably furnish a greater variety of symptoms than all the organic functions combined.

The cranial nerves are nine in number on each side, and are transmitted, through foramina at the base of the skull. They are variable in their size and structure—have a great range of distribution and perform a great variety of functions. They are named numerically according to the order in which they occur, from before, backwards, or pass out of the cranium. Their names are also derived from the part to which they are distributed, or from the special function appropriate to each.

Physiologically or functionally they admit of division, into four groups, namely: nerves of special sense, nerves of common sensation, nerves of motion, and mixed nerves. It is not deemed necessary to arrange the groups in this description in a tabular form; but to notice briefly their superficial origin, direction, distribution, and a part of their functions.

The first cranial nerve, or olfactory, getting its origin from the under and posterior surface of the anterior lobe of the brain, and passing through foramina in the cribriform plate of the ethmoid bone and distributed to the Schneiderian membrane as a special nerve of the sense of smell, gives us the power of distinguishing odors, the various kinds of which convey different impressions to the mind, and, when impaired by disease, is often an important means of diagnosis. The second, or optic, arising from the thalamus and nates of the tubercula quadrigemina, and passing by the name of the optic tract to the olivary process where its commissure is found, from the anterior surface of which each nerve proceeds to its respective foramen opticum, and is exclusively distributed to the eye as a nerve of the special sense of sight. This nerve, together with the third, fourth and sixth, which arise respectively from the inner side of the crura cerebri, from the valve of vieussens, and from the constricted portion of the corpora pyramidalia and passing out of the cranium through the foramen lacerum anterius or sphenoidal fissure to all the muscles of the eye, which they endow with motor influence, and after sending fillaments to the iris, performs the functions of that noble organ which is the master-piece of Nature's works. By this we distinguish objects both far and near, please our fancy of beauty, view armies in battle array, and all the agreeable variety displayed in the landscape of nature; by this sense we perceive the temper and dispositions, the passions and affections of our fellow-creatures, even when they wish to keep them latent; the discerning eye will detect the deception in the countenance; it is the mirror to the soul, the index to the heart, and a stimulant to the will; in disease it furnishes symptoms which are not evinced by any other organ; through it we detect the condition of the brain, foreboding impending evil, or tending to a favorable issue. The fifth *tri-facial* or *iri-geminal* is a mixed nerve, consisting of motor and sensitive fibres and fillaments of the special sense of taste. It is the largest cranial nerve, and resembles a spinal nerve in its origin by two roots and the existence of a ganglion on its posterior root. It extends from the side of the pons varoli to the apex of the petrous portion of the temporal bone, where its sensitive fillaments enter the Casserian ganglion, from the anterior surface of which three large branches proceed, viz: the ophthalmic, the superior and inferior maxillary nerves; they pass out of the cranium respectively through the sphenoidal fissure foramen rotundum and foramen ovale. The first division of the fifth, or ophthalmic, is a sensory nerve, supplying the mucous membrane of the eye and nose, the lachrymal gland and duct, and the integument and muscles of the eyebrow and forehead, making its appearance on the face at the supra-orbital foramen. The power of common sensation being that sense by which we distinguish the different qualities of bodies, such as heat and cold, smoothness from roughness, softness from hardness, solidity, mobility, etc., and the fifth nerve, being the most acutely sensitive nerve in the whole body, it is not surprising that we are quickly admonished of foreign bodies in the eyes and nose. When this irritation is continued, either by foreign bodies, heat or cold, light or injuries, it produces not only excessive local pain, but great constitutional disturbance, with no little amount of febrile action, as is evinced in conjunctivitis and common ophthalmia.

The superior maxillary or second division of the fifth is also a sensory nerve, and is intermediate both in size and position between the ophthalmic and inferior maxillary. It commences at the middle of the Casserian ganglion, and after making its exit through the foramen rotundum, passes across the spheno-maxillary fissure and traverses the floor of the orbit, making its appearance on the face at the infra-orbital foramen. Its branches are numerous, those of the greatest importance supply the teeth and gums of the upper jaw, the soft and hard palate, the tonsils, the antrum of highmore, and the muscles and integument of the lower eyelid, cheek and upper lips.

The third division of the fifth is a compound nerve, consisting of the power of sensation, motion and the special sense of taste. It is the largest of the three divisions, and proceeds from the lower angle of the Casserian ganglion after passing through the foramen ovale it is joined by the anterior or motor root and divides into two trunks, external and internal. The external division consists almost exclusively of fibres from the motor root, and is distributed to all the muscles of mastication. The internal division terminates in three branches, namely: auriculo-temporal, inferior-maxillary and gustatory. These branches, as their names imply, are distributed to the integument of the temple and external ear, the gums and the teeth of the lower jaw, the lower part of the face and lower lip. The gustatory is distributed to the tongue, and is one of the nerves of the special sense of taste. The inferior dental enters the inferior-maxillary at the dental, and makes its exit at the mental foramen. By means of the fifth pair of nerves we are endowed with the greatest pleasure allotted to man, the boon of earth's blessings, the necessities and the luxuries of life, health, happiness, wealth and wisdom, the peculiarities of taste, the desires and imaginations of the mind, the gratification of the will, as evinced by the different senses, are all appeased by the function of this nerve.

On the other hand, when its functions are impaired or vitiated either by disease, habit or injury, it is susceptible of producing equally as great misery as its normal and healthy condition is conducive to happiness. Nay, even more when its special sense of taste together with its concomitant, from the Glosso-pharyngeal is excessively gratified and becomes involved by abuse, it is often culpable of the destruction not only of health, happiness, wealth, integrity, virtue and morality, but even of life itself. Again, when the sensative and motor fibres of this nerve together with the facial are involved by disease it is capable of producing the greatest amount of physical suffering, and the most hideous expressions of the countenance of any of the nerves in the body. This is well exemplified in tic douloureux on the one hand, and hemiplegia of the face on the other.

The seventh pair consists of the facial or portio dura and the auditory or portio mollis. The first or facial is the motor nerve of all the muscles of the face. It extends from the lateral tract of the medulla oblongatta, or from the grove between the olivary and restiform bodies to the meatus auditorius internus which it enters in connection with the auditory nerve After traversing the canal it makes it exit at the stylo-mastoid foramen, and is distributed on the side of the face in a radiated direction under the name of the pes anserinus.

The second division or auditory is the special nerve of the sense of hearing. It arises from the posterior median fissure in the floor of the fourth ventricle winding around the corporia restiformia from which it receives fibres, and entering with the facial the meatus auditorius enternus, and is exclusively distributed to the cochlea vestibula and semicircular canals. By the sense of hearing we distinguish sounds, and are capable of enjoying all the agreeable charms of music—by it we are enabled to receive instruction, discipline the mind, communicate our thoughts and intentions, our wishes and desires, and enjoy the pleasures of society, and by it our reason is rendered capable of exerting its utmost power and energy.

Pathologically it is one of the first to become impaired by disease, is often rendered obtuse, dull and imperfect, and is symptomatic of various conditions, not only of the organ of hearing, but of the nervous centres, as is evinced by an accumulation of cerumen in the meatus or *linnitus aurium* and deafness in fevers, or preceding apoplexy, hysteria and epilepsy.

The eighth pair consists of three nerves, viz: glosso-pharyngeal pneumogastric and spinal accessory. They all arise from the lateral tract of the cord immediately behind the olivary process. The third division or spinal accessory arises by two portions, a vagus and a spinal portion; the latter portion gets its origin as low down as the sixth cervical nerve; the three nerves pass out of the cranium through the jugular foramen. The first division or glosso-pharyngeal, is a compound nerve, and is distributed to the mucous membrane of the fauces and base of the tongue, the mucous glands of the mouth and tonsils; it is the nerve of sensation to the mucous membrane of the pharynx fauces and tonsils; of motion, to the pharyngeal muscles, and a special nerve of taste in all the parts of the tongue to which it is distributed. The second division of pneumogastric is composed of both sensitive and motor fillaments. It has a more extensive distribution than any of the other cranial nerves. It is the nerve of the respiratory organs and the upper part of the alimentary canal, sending branches to the larynx, trachea, lungs, pharynx. œsophagus, stomach and heart. It supplies the organs of the voice and respiration with motor and sensitive fibres, and the pharynx, œsophagus, stomach and heart with motor influence. The third division or spinal accessory, is a nerve of motion, and is distributed to the sterno-mastoid and trapezeus muscles, communicating freely with the cervical nerves. The ninth or hypoylossal arises from the grove between the corpora pyramidalia and corpora olevaria, it emerges through the anterior condyloid foramen, and is distributed to all parts of the tongue as a motor nerve. This nerve is said to consist of both motor and sensitive fillaments in consequence of convulsive movements being produced in the tongue when the fibres of the nerve are irritated in any part of its course. This, however, according to *Longet,* is owing to its inosculations, with sensitive nerves after it emerges from the skull. His experiments having shown conclusively that if it is irritated at its origin it is entirely insensible.

I have thus hastily, briefly, and very imperfectly given the superficial or apparent origin, distribution and functions of the cranial nerves, and have but casually noticed their relations to medicine and surgery.

Under the head of practical remarks it will be necessary to acknowledge the existence of deep fibres or roots of these cerebro-spinal centre nerves, which may in all instances be traced deeply into the superior and inferior cerebral masses, consisting of both white and gray matter; though says *Van Der Konk,* this examination is one of the most difficult investigations in minute anatomy. The peculiar softness of these parts, the fact that they are destroyed by slight pressure, the extraordinary minuteness and delicacy of their tissue, their primative fillaments being quite imperceptible to the naked eye, while it is with difficulty that under a tolerably strong magnifying power, a single thread can be followed even for a very short space.

Practically the brain is divisible into cerebrum, cerebellum, pons-varioli and medulla oblongata. The various diseases and injuries common to these parts are evidenced by the nerves arising from these positions. Through them, in disease we are furnished with very important diagnostic symptoms; by them we are enabled to judge in some instances of the modes of death, particularly of coma paralysis and necramia.

In coma, which signifies that the superior masses or cerebrum is involved, whether from injury or narcotic poison, the first, second, and third nerves are our chief aids in diagnosis, particularly the third, which supplies the iris with motor influence.

If however the cause persists or be increased and is severe, the exito-motory functions become more or less involved. If slight, one pupil may be dilated the other contracted, this condition sometimes exists in apoplexy, and is of very unfavorable import, indicating irritation of the upper portion of the spinal medulla as well as oppression of the superior masses. Irritation of the spinal medulla may produce symptoms as various as its causes are numerous; it may simply produce cerebral excitement manifested by tinnitus aurium, muscæ volitantes, numbness and tingling sensations in the limbs, loss of memory, confusion of ideas, hallucinations, or in its usual signs of intoxication, and in delirium or convulsions, vomiting, hiccough, contracted pupil, etc.

This is often the case when the narcotic poisons have been administered, such as conium, belladonna, alcohol in large quantities, carbonic acid, ether, chloroform introduced by inhalation, and sometimes the excrementitious matter in the blood and bile. Opium and its preparations sometimes produce the same effects before destroying the functions of the superior masses of the nervous system. But if that portion termed by Sir Charles Bell, the respiratory tract be involved sufficiently to suspend the functions of the respiratory nerves, death occurs instantaneously from paralysis of the second division of the eighth pair or vagus, which supplies the lungs and heart with motor influence. If, however, portions of the medulla oblongata be simply irritated sufficiently to produce reflex action, various physiological changes are produced which are peculiar to themselves.

This is well exemplified by irritation of the floor of the fourth ventricle, which increases the glycogenic function of the liver to such an extent that diabetic urine is the result. Concerning the organic changes there is another condition which exists and of a chronic character, involving portions of the encephalic mass from which the deep fibres of the cranial nerves emerge. It has become an established fact that in those who have by disease of the brain, lost their speech, the corpora olivaria is found in a softened, broken down, disintegrated condition, and in those who are congenitally dumb, those bodies are very much atrophied. The same can be said of the corpora restiformia in congenital deafness the deep fibres of the auditory nerve being traced into the ganglionic cells of this body.

Again, in strabismus convergens, produced by repeated convulsions, the upper portion of the corpora pyramidalia is found materially changed, this being the origin of the abducens, or sixth nerve, which supplies the external rectus muscle with motor fillaments. Many more pathological conditions might be enumerated, showing the anatomical relations of the *cranial nerves* to *medicine* and *surgery*, but time will not permit. I will therefore submit these feeble suggestions for your consideration, trusting your acquiescence of the necessity of a more thorough knowledge of *Practical Anatomy.*

